# Supplementary material for: Exploring the utility of dynamic motor control to assess recovery following pediatric traumatic brain injury: A pilot study
Source: PLoS One. 2026 Feb 4;21(2):e0322674. doi: 10.1371/journal.pone.0322674 (PMC12871996; doi:10.1371/journal.pone.0322674)
Supplement: S2 File — (DOCX) [file pone.0322674.s002.docx]

Caregiver Report Questionnaire

Participants Name: _________________________

Date of Visit: ______________________________

We would like to know how your child is doing after their traumatic brain injury. Please pick/circle the statement that best describes your child.

1. My child’s mobility is…
2. A lot worse than before the injury
3. A little worse than before the injury
4. Not better or worse than before the accident
5. A little better than before the injury
6. A lot better than before the injury
7. My child’s memory is...
8. A lot worse than before the injury
9. A little worse than before the injury
10. Not better or worse than before the accident
11. A little better than before the injury
12. A lot better than before the injury
13. My child’s mood is...
14. A lot worse than before the injury
15. A little worse than before the injury
16. Not better or worse than before the accident
17. A little better than before the injury
18. A lot better than before the injury
